# Antimicrobial Resistance and Species Dynamics of *Enterococcus faecalis* and *Enterococcus faecium* in Breeding Hens: Phenotypic and Genotypic Characterization

**DOI:** 10.3390/vetsci13080795

**Published:** 2026-08-09

**Authors:** Alejandro Fenollar, María A. Ferrús, Pablo Catalá-Gregori, Vicente Tallá-Ferrer, Miguel García-Ferrús, Ana Isabel Jiménez-Belenguer

**Affiliations:** 1 Biotechnology Department, Universitat Politècnica de Valencia. Cno. Vera s.n., 46022 Valencia, Spain; alejandro.fpenades@gmail.com; 2 Centro Avanzado de Microbiología Aplicada, Universitat Politècnica de Valencia, Cno. Vera s.n., 46022 Valencia, Spain; migarfe1@upv.es (M.G.-F.); anjibe@upvnet.upv.es (A.I.J.-B.); 3 Centro de Calidad Avícola y Alimentación Animal de la Comunidad Valenciana (CECAV), CEU Universities, Universidad CEU Cardenal Herrera, 46115 Alfara del Patriarca, Spain; p.catala@cecav.org; 4 Grupo SADA, Ronda de Poniente 9, 28760 Tres Cantos, Spain; vtalla@sadavc.es

**Keywords:** *Enterococcus faecalis*, *Enterococcus faecium*, antimicrobial resistance, breeding hens, poultry, tetracycline, erythromycin, resistance genes, One Health, food safety

## Abstract

*Enterococcus faecalis* and *Enterococcus faecium* are commensal bacteria of poultry but also important opportunistic pathogens and key indicators for antimicrobial resistance (AMR) surveillance within a One Health framework. However, data on the occurrence and antimicrobial resistance of enterococci in breeding hens remain limited despite their critical role in the poultry production pyramid. This study evaluated the prevalence, antimicrobial resistance profiles, and resistance genes of *Enterococcus faecalis* and *Enterococcus faecium* isolated from two commercial breeding hen farms in eastern Spain. A total of 330 isolates were obtained from different production stages and sample types. Species identification was performed by multiplex PCR, antimicrobial susceptibility was assessed using disk diffusion according to Clinical and Laboratory Standards Institute (CLSI) guidelines, and resistance genes (*erm* and *tet*) were detected by PCR. A significant age-related shift from *E. faecalis* in early production stages to *E. faecium* in adult hens was observed. High resistance rates were detected for tetracycline (up to 95.2%) and erythromycin, whereas resistance to critically important antimicrobials such as vancomycin, aminoglycosides, and ampicillin was rare or absent. Significant differences between farms were identified in both antimicrobial resistance levels and the prevalence of multidrug resistance. The most prevalent resistance genes were *ermB*, *tetL*, and *tetM*, frequently co-occurring. This study shows that breeding hens constitute a relevant reservoir of AMR within poultry production systems. Species dynamics and resistance patterns appear to be influenced by age and farm-related factors. These findings underscore the need for continuous AMR surveillance integrating phenotypic and genotypic approaches from a One Health perspective.

## Simple Summary

Antibiotic resistance is a growing global concern affecting both human and animal health. Resistant bacteria that naturally inhabit animals can spread through the food chain or the environment. This study focused on two common bacterial species in breeding chickens, *Enterococcus faecalis* and *Enterococcus faecium*, which can act as pathogens in both animals and humans and are important carriers of antibiotic resistance. The research analysed their prevalence, antibiotic resistance, and the resistance genes they harbour in hens of different ages from two farms in Spain. Results showed that while *E. faecalis* predominated in young chicks, *E. faecium* became dominant in adult birds. High resistance was detected to antibiotics widely used in poultry, while resistance to those critical for human medicine was rare or absent. Differences between farms were also observed, indicating that age and management practices may influence resistance patterns, and thus highlighting the need for continuous monitoring in poultry production to help protect animal health, ensure food safety, and reduce the risk of spreading resistance to humans.

## 1. Introduction

*Enterococcus* spp. are Gram-positive bacteria widely distributed in the gastrointestinal microbiota of humans and animals, as well as in environmental reservoirs. Due to their persistence and faecal origin, they are commonly used as indicators of contamination and antimicrobial resistance (AMR) surveillance [1]. At the same time, enterococci are opportunistic pathogens of increasing relevance, particularly *Enterococcus faecalis* and *Enterococcus faecium*, which are among the most frequently isolated species from animal samples [2] and are frequently associated with severe infections in humans, including septicaemia, endocarditis, and urinary tract infections [3]. In poultry production systems, enterococci are part of the normal intestinal microbiota but can also act as opportunistic pathogens, contributing to conditions such as septicaemia or skeletal disorders, with potential economic impact on flock productivity [4]. In addition to their veterinary relevance, *Enterococcus* spp. are considered key indicator bacteria for monitoring AMR in food-producing animals [1] because they exhibit intrinsic resistance to several antibiotics and are able to acquire additional resistance through plasmid and transposon transfer, as well as chromosomal mutations [3]. Antibiotic-resistant enterococci have been isolated worldwide from both poultry and poultry products [5,6].

The poultry industry represents one of the largest and fastest-growing sectors of animal protein production worldwide [7]. The use of antimicrobials for therapeutic, prophylactic, and sometimes production-related purposes has contributed to the emergence and dissemination of antimicrobial resistance in poultry-associated bacteria. Tetracyclines and macrolides have historically been widely used, and resistance to these antimicrobial classes remains highly prevalent in enterococci isolated from poultry systems [3,5,8]. From a veterinary perspective, the presence of antimicrobial-resistant enterococci in poultry production is particularly relevant as these bacteria can colonize animals throughout their life cycle, persist in the farm environment, and spread within and between flocks. Moreover, management practices, housing systems, and feeding strategies can influence both gut microbiota composition and the selection of resistant strains [9]. From a One Health perspective, antimicrobial resistance in enterococci from poultry is a matter of veterinary and public health concern, as resistant bacteria and resistance genes may be transmitted to humans through the food chain, direct contact, or the environment [1,10]. Recent reviews emphasize that poultry production is a key interface where antimicrobial resistance can emerge and disseminate, requiring integrated surveillance strategies across sectors [11].

Despite the large body of research on antimicrobial resistance in broilers, comparatively limited information is available on breeding and laying hens, especially under commercial production conditions [12,13]. This lack of data represents a significant knowledge gap, as breeding and laying hens occupy a critical position in the poultry production pyramid and may act as reservoirs of antimicrobial resistance that can be transmitted vertically or horizontally across the production chain [14,15]. Therefore, the aim of this study was to investigate the prevalence of antimicrobial resistance and the distribution of macrolide and tetracycline resistance genes in *Enterococcus faecalis* and *Enterococcus faecium* isolated from breeding hens in commercial poultry farms in Spain, contributing to the understanding of antimicrobial resistance within the poultry production pyramid and its implications under a One Health framework.

## 2. Materials and Methods

### 2.1. Sampling

Two breeding hen farms (A and B) located in eastern Spain were included in the study. All animals belonged to the same breeder line and originated from the same breeder flock, hatchery, and production batch. Farms A and B were part of the same production system. Farm A corresponded to the rearing farm, where birds were raised from day-old chicks until 28 weeks of age. Subsequently, these birds were transferred to Farm B, which served as the production farm.

In farm A, a flock of 14,500 hens (*Gallus gallus*) was sampled at five time points: upon arrival at the farm (sampling SA1; one-day-old chicks), during the rearing phase (samplings SA2 and SA3; 4- and 19-week-old pullets), and during the production phase (SA4 and SA5; 25- and 28-week-old hens) (Table 1). During the study period, tylosin and amoxicillin were administered for therapeutic purposes at week 4 (5-day treatment) and week 25 (4-day treatment), respectively.

In Farm B, the flock consisted of 4100 adult breeder hens and 408 males. Samples were collected at ten time points at three-week intervals (SB1–SB10). Birds were 28 weeks old at the first sampling and 54 weeks old at the last one (Table 1). A treatment with enrofloxacin for 5 days was administered by authorized veterinarians at the SB4 sampling point (36-week-old animals).

The samples were collected in accordance with the guidelines of the “National Programme for the surveillance and control of certain *Salmonella* serotypes in flocks of breeding hens of the species *Gallus gallus* [16], to ensure that the samples were representative of the entire flock.

Sampling SA1 consisted of four 25 g samples collected from transport boxes containing meconium from one-day-old chicks. All other samplings in both farms consisted of a pair of absorbent boot swabs impregnated with the soil residues of the farm and two composite faecal samples (approximately 100 g of manure collected from belt systems) (Table 1). All samples were refrigerated and processed within 24 h.

Samples were diluted in buffered peptone water (Buffered Peptone Water, ISO, Scharlau, Barcelona, Spain). In SA1, 25 g of sample were mixed with 225 mL of peptone water. Boot swabs were weighed and mixed in 1:10 *w*/*v* peptone water. For composite faecal samples, 25 g were taken, diluted and homogenized in 225 mL of peptone water using a stomacher (BagMixer, Interscience, Paaris, France).

### 2.2. Isolation and Identification of Enterococcus spp.

After homogenization, serial decimal dilutions were prepared and plated on Slanetz–Bartley agar (Scharlab S.L., Barcelona, Spain). For sampling SA1, 0.1 mL aliquots were spread onto 30 plates, whereas for the rest of the samplings, 0.1 mL aliquots were spread onto 20 plates per type of sample (boot swabs or faeces). Plates were incubated at 37 °C for 48 h, and colonies displaying typical *Enterococcus* colour and morphology were randomly selected, with one colony chosen from each plate and streaked onto Brain Heart Infusion Agar (BHIA, Scharlab S.L., Barcelona, Almeería, Spain).

Thirty colonies were selected at SA1, whereas up to 15 colonies per sample type were selected for the remaining sampling points across both farms. After incubation for 24 h at 37 °C, isolates were further confirmed by incubation for 2 h at 42 °C on kanamycin–aesculin–azide agar (KAA agar, Thermo Fisher Scientific, Basingstoke, UK), selecting colonies showing a black halo surrounding growth.

Confirmed *Enterococcus* spp. isolates were identified as *E. faecalis* or *E. faecium* by multiplex PCR targeting the *ddl* gene, which encodes D-Ala–D-Ala ligase, using primers and conditions described by Depardieu et al. [17] (Table 2).

Briefly, the mPCR assays were carried out in a final volume of 25 µL with the following reagent concentrations: 1× NH_4_ Reaction Buffer (BIOTAQ™ DNA Polymerase, Bioline, Almería, Spain), 0.5 mM of each dNTP (DNTP Mix 100 mM, Bioline, Almería, Spain), 1.5 mM MgCl_2_ (BIOTAQ™ DNA Polymerase, Bioline, Almería, Spain), 1.25 U Taq DNA polymerase (BIOTAQ™ DNA Polymerase, Bioline Almería, Spain), and the primer concentrations as specified by the authors. Primers were designed by TIB MOLBIOL (Berlin, Germany). Amplification was carried out in a SimpliAmp™ Thermal Cycler (Applied Biosystems, Waltham, MA, USA) as follows: 3 min at 94 °C and 30 cycles of amplification consisting of 1 min at 94 °C, 1 min at 54 °C, and 1 min at 72 °C, with 7 min at 72 °C for the final extension. In all reactions, a negative control in which DNA was replaced with 2.5 μL of sterile ultrapure water was used as a negative control. For positive control, *E. faecalis* ATCC 29212 and *E. faecium* ATCC 19434 were used.

PCR products (15 μL) were visualized by electrophoresis on a 2.5% (*w*/*v*) agarose gel (Pronadisa, Maddrid, Spain) in Tris-acetate-EDTA (TAE) buffer (PanReac AppliChem, Barcelona, Spain) containing 0.005% RedSafe (iNtRON Biotechnology, Seongnam, Republic of Korea), at 90 V for 2 h and viewed under UV transillumination. A 100 bp molecular weight marker (Thermo Fisher Scientific, Waltham, MA, USA) was used.

Confirmed isolates were stored at −21 °C in cryovials (Pro-lab Diagnostics MicrobankTM, Richmond Hill, ON, Canada) until further analysis.

### 2.3. Antimicrobial Susceptibility Testing

Antimicrobial susceptibility was determined using the disk diffusion method (antibiotic disks, Oxoid Ltd., Basingstoke, UK) on Mueller–Hinton agar (Scharlau, Barcelona, Spain), following Clinical and Laboratory Standards Institute (CLSI M100-S25) guidelines [18]. Plates were incubated at 37 °C for 18 h.

The antibiotics tested were selected according to European Food Safety Authority (EFSA) recommendations [19]: erythromycin (15 µg), vancomycin (30 µg), gentamicin (120 µg), streptomycin (300 µg), quinupristin/dalfopristin (15 µg), ampicillin (10 µg), tetracycline (30 µg), chloramphenicol (30 µg), and ciprofloxacin (5 µg). High-content aminoglycoside disks were used to detect high-level resistance, as enterococci exhibit intrinsic low-level resistance and may appear susceptible in vitro while requiring higher therapeutic doses in vivo [18,20]. *E. faecalis* was not tested for quinupristin/dalfopristin because of its intrinsic resistance [2,19].

Antimicrobial susceptibility results were interpreted according to the CLSI M100-S25 guidelines [18]. Isolates were classified as susceptible, intermediate, or resistant based on the species-specific clinical breakpoints established for *Enterococcus* spp. For statistical analyses, isolates categorized as intermediate were considered non-resistant.

Resistance levels were classified according to EFSA and European Centre for Disease Prevention and Control (ECDC) [1] as follows: rare (<0.1%), very low (0.1–1.0%), low (>1–10%), moderate (>10–20%), high (>20–50%), very high (>50–70%), and extremely high (>70%).

### 2.4. Molecular Analysis

DNA extraction was performed using the GenElute Bacterial Genomic DNA Kit (Sigma-Aldrich, St. Louis, MO, USA). DNA samples were stored at −21 °C.

All the isolates resistant to erythromycin and tetracycline were tested for the presence of *tet* and MSL-related genes. The genes analysed were *erm*A and *erm*B for MLS resistance, and *tet*K, *tet*L, *tet*M, *tet*O, and *tet*S for tetracycline resistance [21,22].

Amplifications were performed in a SimpliAmp™ Thermal Cycler (Applied Biosystems, Waltham, MA, USA) described by Chen et al. [21], while tetracycline gene amplification followed the Ng et al. [22] protocol. Reactions were performed in a final volume of 25 µL containing 1× NH_4_ reaction buffer (BIOTAQ™ DNA Polymerase, Bioline), 0.5 mM dNTPs (DNTP Mix 100 mM, Bioline), MgCl_2_ (2.5 mM for MLS and 3 mM for tetracycline genes) (BIOTAQ™ DNA Polymerase, Bioline), 1.25 U Taq DNA polymerase (BIOTAQ™ DNA Polymerase, Bioline), and primers (synthesized by TIB MOLBIOL, Berlin, Germany) at the concentrations specified in Table 3. Positive controls (DNA from isolates carrying each resistance gene and previously characterized in our laboratory) and negative controls (sterile ultrapure water) were included in all PCR reactions.

### 2.5. Statistical Analysis

Statistical analyses were made using “Statgraphics Centurion XVII.II” (Statpoint Technologies Inc., Warrenton, VA, USA) software, using the isolate-level data presented in Supplementary Table S1. Categorical variables (species distribution, antimicrobial resistance, resistance profiles, and gene prevalence) were compared using a χ^2^ test or Fisher’s exact test when expected frequencies were low. A non-parametric Mann–Whitney U test was used for comparisons of the number of resistances per isolate. To account for multiple comparisons, Bonferroni correction was applied. Statistical significance was set at *p* < 0.05.

## 3. Results

### 3.1. Isolation of Enterococcus spp.

Colonies exhibiting the typical morphology of *Enterococcus* spp. on Slanetz–Bartley agar, producing a black halo on AAK agar, and testing positive by PCR with the species-specific primers described by Depardieu et al. [17] were identified as *Enterococcus faecalis* or *Enterococcus faecium* (Table S2). A total of 150 isolates were identified from Farm A and 180 from Farm B (Table 4).

A significant difference in species distribution was observed between farms (χ^2^ test, *p* = 0.00001), with higher odds of isolating *E. faecalis* in Farm A (OR = 3.65, 95% CI: 2.08–6.40). When analysed by age group, the distribution of species also varied significantly across production stages. In Farm A, all isolates recovered from one-day-old chicks were identified as *E. faecalis* (100%), showing a significantly higher prevalence of this species than both pullets (OR = 236.7, 95% CI: 13.5–4144.2, *p* < 0.0001) and adult hens (OR = 615.5, 95% CI: 32.9–11511.2, *p* < 0.0001).

The proportion of *E. faecium* in Farm A increased with age, representing 80% of isolates in pullets and 91.7% in adult hens. A similar pattern was observed in adult hens from Farm B, where *E. faecium* was also the predominant species (88.9%). No significant differences in species distribution were observed between pullets and adult hens from Farm A or between adult hens from farms A and B.

### 3.2. Antimicrobial Resistance

The full distribution of resistance percentages is shown in Table 5 and detailed in Supplementary Material (Table S1). Isolates from both farms were mainly resistant to tetracycline and, to a lesser extent, erythromycin.

In *E. faecalis* isolates from Farm A, extremely high resistance levels were detected for tetracycline and very high levels for erythromycin, ciprofloxacin and chloramphenicol. In *E. faecalis* isolates from Farm B, very high resistance to tetracycline and low resistance to erythromycin were detected, while resistance to other antibiotics was absent. No isolate from either farm was resistant to ampicillin, vancomycin, gentamicin or streptomycin.

*E. faecium* isolates from Farm A showed extremely high resistance to tetracycline, very high resistance to erythromycin, low resistance to quinupristin/dalfopristin and ciprofloxacin, and very low resistance to ampicillin. In Farm B, extremely high resistance to tetracycline, moderate resistance to erythromycin, and very low resistance to ciprofloxacin and quinupristin/dalfopristin were observed.

Significant differences between farms and species were observed for some antibiotic resistance phenotypes (Supplementary Material, Table S4). Among *E. faecalis* isolates, resistance to erythromycin was significantly more frequent in Farm A than in Farm B (59.6% vs. 10.0%; OR = 13.26, 95% CI: 2.75–63.93; *p* = 0.00016). Likewise, ciprofloxacin resistance was detected exclusively in Farm A (59.6% vs. 0%; OR = 58.95, 95% CI: 3.36–1035.58; *p* < 0.0001). In contrast, no significant differences in tetracycline resistance were observed between farms. Among *E. faecium* isolates, Farm A exhibited significantly higher resistance rates than Farm B for all antimicrobials evaluated.

When both species were considered together (Supplementary Material, Table S4), significant differences were observed for erythromycin, ciprofloxacin and chloramphenicol resistance, with significantly higher rates in Farm A compared to Farm B (*p* < 0.001 in all cases), whereas tetracycline resistance did not differ significantly between farms.

### 3.3. Antimicrobial Resistance Profiles of Isolates

Resistance profiles were determined for all isolates exhibiting at least one resistance (Table 6).

Among *E. faecalis* isolates from Farm A, 75.7% were resistant to four antibiotics, whereas in Farm B, most isolates were resistant to either one antibiotic or none. For *E. faecium*, most isolates in Farm A were resistant to one or two antibiotics, while in Farm B, the majority showed resistance to only one antibiotic. In both farms, resistance to more than three antibiotics was rare. The number of antimicrobial resistances per isolate differed significantly between farms for both species, with significantly higher values observed in Farm A (*p* < 0.001) (Table S1).

Tetracycline resistance was the most common trait in all resistance profiles, frequently detected either alone or in combination with erythromycin resistance. Only two resistance profiles were identified among *E. faecalis* isolates across both farms. In Farm A, the most common profile was E-TE-C-CIP, whereas in Farm B the predominant profile was TE alone. Among *E. faecium* isolates, six profiles were observed in Farm A, with E–TE and TE being the most frequent, while in Farm B, five profiles were identified, with TE alone being the most frequent pattern.

Multidrug resistance (MDR), defined as resistance to three or more antimicrobial classes [23], was detected in 75.7% of *E. faecalis* isolates in Farm A, whereas no MDR isolates were found in Farm B. In *E. faecium*, MDR prevalence was lower (12.6% in Farm A and 1.3% in Farm B).

### 3.4. Antibiotic Resistance Genes

The prevalence of resistance genes detected in isolates resistant to erythromycin and tetracycline is shown in Table 7. Among MLS resistance genes, *ermB* was the most prevalent in both farms, being detected in all *E. faecalis* isolates and in 94.8% and 34.6% of *E. faecium* isolates in farms A and B, respectively. The *ermA* gene was detected only in *E. faecalis* from Farm A.

For tetracycline resistance, *tet*K, *tet*L, and *tet*M were the most prevalent genes. *tet*L and *tet*M were present in all *E. faecalis* isolates from Farm A, while in Farm B, *tet*K, *tet*L, *tet*M, and *tet*O were also detected. In *E. faecium* isolates, *tet*K, *tet*L, and *tet*M were the most frequently detected genes in both farms.

Multiple resistance genes commonly co-occurred within the same isolates, with profiles including *erm*B-*tet*L-*tet*M being the most predominant (Table S1). Both in farms A and B, *E. faecalis* isolates carrying more than one gene showed a reduced diversity of profiles, with nearly all strains carrying the combination *erm*A-*erm*B-*tet*L-*tet*M (Farm A) and *erm*B-*tet*K-*tet*L-*tet*M (Farm B). *E. faecium* displayed a greater diversity of profiles, with a lower predominance of *erm*B-associated profiles and a higher proportion of tetracycline-only gene combinations in Farm B.

Taken together, gene distribution differed significantly between farms (*p* < 0.001), mainly due to differences in the prevalence of *erm*A (36.0% in Farm A vs. 0% in Farm B), *erm*B (markedly more frequent in Farm A, OR = 42.76, 95% CI: 10.1–181.2), and *tet*K (significantly more prevalent in Farm B, OR = 0.33, 95% CI: 0.12–0.92). When data from both farms were pooled, comparisons revealed significant differences between species for *erm*B and *tet*K (Supplementary Material, Table S4).

Significant differences were observed in the prevalence of resistance genes among *E. faecium* isolates from Farm A and Farm B (Supplementary Material, Table S4). The prevalence of *ermB* was markedly higher in Farm A than in Farm B (OR = 34.63, 95% CI: 8.41–142.60). Likewise, *tetM* was significantly more common in Farm A (OR = 8.15, 95% CI: 1.95–34.04). In contrast, no significant differences were detected for *tetK* or *tetL*. For *E. faecalis*, meaningful statistical comparisons could not be performed because the number of resistant isolates available from Farm B was very limited (*n* = 2).

## 4. Discussion

This study provides new insights into antimicrobial resistance patterns in Enterococcus faecalis and Enterococcus faecium from breeding hens, an understudied stage in the poultry production pyramid. A key finding is the age-related shift from *E. faecalis* in early stages to *E. faecium* in adult hens. This ecological shift was supported by the statistical analyses, with differences observed between age groups within Farm A and between farms, suggesting that host age and production stage may play an important role in shaping enterococcal populations. However, as we did not perform a longitudinal study tracking the same animals throughout the entire production cycle in both farms, our findings should be interpreted as temporal changes across production stages rather than true longitudinal dynamics at the individual level.

Experimental studies have shown that antimicrobial exposure, including tylosin, may promote a shift in enterococcal populations towards *E. faecium*, likely due to its higher intrinsic and acquired resistance capacity [24]. Moreover, recent studies indicate that *E. faecium* has greater ecological adaptability in intensive systems, including stress tolerance and an enhanced capacity to acquire resistance determinants [25]. Its predominance in adult hens may therefore reflect both resistance and ecological fitness advantages, as also observed in European systems [5,8]. This shift has important One Health implications, since *E. faecium* is a major reservoir of antimicrobial resistance genes and is frequently associated with nosocomial infections [26]. Its dominance in adult birds increases the epidemiological relevance of poultry as a resistance reservoir.

High resistance rates were observed for tetracyclines, in agreement with [27], reinforcing its role as a key driver of resistance selection in poultry. Despite reduced antimicrobial use in Europe, surveillance studies indicate that tetracycline resistance remains among the highest [28]. Similar patterns have been reported in other Spanish poultry systems [5].

An interesting finding was that ciprofloxacin resistance was detected only in isolates from Farm A, even though enrofloxacin treatment was reported only in Farm B. Although the present study does not allow causal inferences, several explanations may account for this observation. Previous studies have shown that fluoroquinolone-resistant bacteria can persist in poultry production systems through the introduction of colonized day-old chicks or through environmental reservoirs, even in the absence of direct fluoroquinolone administration [29]. Therefore, the detection of ciprofloxacin-resistant isolates exclusively in Farm A should not be interpreted as indicating a direct association between current antimicrobial use and the observed resistance pattern. Rather, it may reflect the persistence of resistant strains acquired earlier in the production chain, historical selective pressures, or other farm-specific ecological factors.

Resistance to clinically relevant antibiotics such as vancomycin, high-level aminoglycosides, streptomycin and ampicillin was rare or absent, consistent with previous reports of lower resistance in animal isolates than in human isolates [30]. In Spain, resistance to vancomycin and chloramphenicol in *E. faecium* has been reported as rare, although higher resistance to quinupristin/dalfopristin has been described elsewhere, differing from our results [5,12]. For *E. faecalis*, moderate or high resistance to streptomycin has also been previously reported [30,31], while in our study, no resistance was detected. The absence of these resistances is consistent with the limited use of these antimicrobials in poultry production and suggests a relatively low selective pressure for these antibiotics in this context.

Despite the administration of some short-term antimicrobial treatments such as tylosin and amoxicillin, no noticeable increase in resistance levels was observed after them. This finding seems to indicate that short-term therapeutic exposure may not be sufficient to significantly alter resistance patterns in enterococci populations, as suggested by other authors [32,33].

Multidrug resistance (MDR) levels were lower than in previous studies [30,34]. This limited accumulation of resistance traits is also consistent with relatively limited selective pressure. When present, MDR almost always involved tetracycline, often combined with erythromycin. The frequent co-occurrence of tetracycline and erythromycin resistance suggests co-selection mechanisms [35]. Interestingly, MDR prevalence was higher in *E. faecalis* than in *E. faecium* in Farm A, contrasting with previous reports where *E. faecium* is often the primary MDR species [36,37,38].

To identify the genetic determinants associated with the observed phenotypic resistance, all the isolates resistant to erythromycin and tetracycline were tested for the presence of *tet* and MSL-related genes. The predominance of *erm*B, *tet*L, and *tet*M agrees with genomic studies identifying these as widespread resistance determinants and describing their association with mobile elements such as plasmids and transposons [8], thus facilitating horizontal transfer and persistence even without antimicrobial pressure and increasing dissemination potential along the food chain.

Multiple resistance genes were frequently detected within single isolates, even with limited MDR, particularly combinations involving *erm*B, *tet*L, and *tet*M, indicating the widespread presence of stable resistance gene clusters and further supporting the role of co-selection [35].

The frequent co-detection of *tet*L and *tet*M is particularly noteworthy, as it indicates the coexistence of different resistance mechanisms (efflux pumps and ribosomal protection), potentially increasing resistance stability under varying environmental conditions. These findings reinforce the importance of monitoring not only phenotypic resistance but also the underlying genetic determinants [36].

Comparison between farms indicated distinct gene distribution patterns. Farm A showed a higher prevalence and diversity of resistance genes, particularly among *E. faecalis*, in which *erm*A, *erm*B, *tet*L, and *tet*M were detected in 100% of isolates. In contrast, Farm B exhibited a reduced gene spectrum, especially for *E. faecium*. Given that bird age and farm were intrinsically linked in the present study, it was not possible to determine whether these differences were associated with age-related changes, farm-specific conditions, or a combination of both factors.

Taken together, the distribution of *Enterococcus* species and the resistance burden appeared to differ between the two studied farms. Isolates from Farm A, which included younger birds, tended to show higher resistance levels, both in terms of prevalence and number of resistances per isolate. This trend was also reflected in the analysis of resistance counts per isolate, which yielded higher values in Farm A for both species. In addition, resistance to several antibiotics, including ciprofloxacin and chloramphenicol, was detected exclusively in Farm A. Although statistical differences were identified, these findings should be interpreted with caution given the potential clustering of isolates within farms and samples. These variations are also likely influenced by differences in bird age and production stages rather than consistent farm-specific factors. Therefore, caution is required when attributing these differences to management or environmental conditions alone.

In summary, these findings are consistent with the hypothesis that younger birds may initially carry a higher load of antimicrobial resistance determinants, which may decrease over time in the absence of sustained selective pressure. This interpretation is consistent with previous studies showing that newly hatched chicks can harbour bacteria with high levels of antimicrobial resistance. *E. coli* isolated from the meconium of one-day-old chicks has been shown to exhibit high resistance rates [14,39], supporting the idea that antimicrobial resistance may be introduced early in the production chain. Such early acquisition of resistance may occur through vertical transmission from parent flocks or through contamination during hatching. Proposed mechanisms include infection of the reproductive tract during egg formation or faecal contamination at oviposition, as well as transmission at hatchery level [14,40,41]. In this context, although the source of colonization could not be determined in the present study, the higher resistance levels observed in Farm A are consistent with the persistence of antimicrobial-resistant enterococci acquired early in life, which could progressively decline as birds age and are no longer exposed to the same selective pressures.

Our findings suggest that the antimicrobial resistance burden in breeding hens’ farms may be influenced not only by immediate antimicrobial use but also by the temporal dynamics of resistance acquisition and loss throughout the production cycle. Variations in antimicrobial practices, biosecurity measures, or environmental conditions may further contribute to these patterns, although such factors were not directly assessed in this study. The results of this work emphasize the importance of considering bird age and antibiotic exposure together with environmental or production factors when interpreting antimicrobial resistance patterns in poultry production systems.

Several limitations should be considered when interpreting our findings. Sample size imbalance between farms, particularly for *E. faecalis* in Farm B, may have reduced statistical power. Additionally, although multiple sampling points were included, isolates originated from a limited number of samples collected on only two farms, which may introduce potential clustering effects, and therefore cannot be considered fully independent observations. Thus, the statistical significance of some comparisons should be interpreted with caution, since they may have been overestimated. An additional methodological consideration is that, although the antimicrobial panel was selected according to EFSA recommendations, susceptibility results were interpreted using CLSI M100-S25 clinical breakpoints rather than EUCAST epidemiological cut-off values (ECOFFs), which are commonly applied in European AMR surveillance. This approach was chosen to characterize phenotypic resistance patterns and their potential public health relevance. However, because clinical breakpoints and ECOFFs may classify some isolates differently, direct comparisons with studies following the EFSA/EUCAST framework should be interpreted with caution. Future studies including a larger number of farms and accounting for the hierarchical structure of the data would help to confirm the robustness and generalizability of the observed patterns. In addition, further research should focus on the genomic characterization of resistance determinants, the role of mobile genetic elements in their dissemination, and the influence of specific farm management practices on the selection, maintenance, and spread of antimicrobial resistance. Future studies could also compare resistance estimates obtained using clinical breakpoints and EUCAST epidemiological cut-off values to facilitate harmonization and comparability with European antimicrobial resistance surveillance programmes.”

## 5. Conclusions

This study suggests that antimicrobial resistance in *E. faecalis* and *E. faecium* from breeding hens is generally low at the phenotypic level, except for tetracycline and erythromycin. However, the widespread presence and co-occurrence of resistance genes indicate a substantial potential for persistence and dissemination of antimicrobial resistance. The observed species shift across production stages and the stability of resistance patterns reinforce the importance of including breeding hens in AMR surveillance strategies. These findings support a One Health approach and underscore the need to integrate phenotypic and genotypic data to better understand and control antimicrobial resistance within poultry production systems.

## Figures and Tables

**Table 1:** Sampling, type and number of samples.

Farm	Sampling	Age of Animals	Sample Type	Nº of Isolates
A	SA1	One-day-old chicks	Meconium (4 samples)	7
				7
				7
				9
	SA2	4-week-old pullets	faeces	15
			boot swabs	15
	SA3	19-week-old pullets	faeces	15
			boot swabs	15
	SA4	25-week-old hens	faeces	15
			boot swabs	15
	SA5	28-week-old hens	faeces	15
			boot swabs	15
B	SB1	28-week-old hens	faeces	10
			boot swabs	10
	SB2	30-week-old hens	faeces	10
			boot swabs	10
	SB3	33-week-old hens	faeces	5
			boot swabs	5
	SB4	36-week-old hens	faeces	5
			boot swabs	5
	SB5	39-week-old hens	faeces	10
			boot swabs	10
	SB6	42-week-old hens	faeces	10
			boot swabs	10
	SB7	45-week-old hens	faeces	10
			boot swabs	10
	SB8	48-week-old hens	faeces	10
			boot swabs	10
	SB9	51-week-old hens	faeces	10
			boot swabs	10
	SB10	54-week-old hens	faeces	10
			boot swabs	10

**Table 2:** Primers used for the species identification of *Enterococcus* spp. isolates.

Primers	Specie	Sequence	Primers Concentration	Size (bp)	Reference
DD13(+)	*E. faecalis*	CACCTGAAGAAACAGGC	0.4 µM	475	(Depardieu et al., 2004) [17]
DD3-2(−)		ATGGCTACTTCAATTTCACG	0.4 µM		
FAC1-1(+)	*E. faecium*	GAGTAAATCACTGAACGA	0.4 µM	1091	
FAC2-1(−)		CGCTGATGGTATCGATTCAT	0.4 µM		

**Table 3:** Primers used for the antibiotic resistance genes to tetracyclines and MLS.

Primers	Sequence	Primer Concentration	Size (bp)	Reference
erm(A)-106f	GAA ATY GGR TCA GGA AAA GG	0.5 µM	332	(Chen et al., 2007) [21]
erm(A)-437r	AAY AGY AAA CCY AAA GCT C	0.5 µM		
erm(B)-91f	GAT ACC GTT TAC GAA ATT GG	0.5 µM	364	
erm(B)-454r	GAA TCG AGA CTT GAG TGT GC	0.5 µM		
tetK-f	TCG ATA GGA ACA GCA GTA	1.25 µM	169	(Ng et al., 2001) [22]
tetK-r	CAG CAG ATC CTA CTC CTT	1.25 µM		
tetM-f	GTG GAC AAA GGT ACA ACG AG	0.5 µM	406	
tetM-r	CGG TAA AGT TCG TCA CAC AC	0.5 µM		
tetL-f	TCG TTA GCG TGC TGT CAT TC	1 µM	267	
tetL-r	GTA TCC CAC CAA TGT AGC CG	1 µM		
tetO-f	AAC TTA GGC ATT CTG GCT CAC	1.25 µM	515	
tetO-r	TCC CAC TGT TCC ATA TCG TCA	1.25 µM		
tetS-f	CAT AGA CAA GCC GTT GAC C	0.5 µM	667	
tetS-r	ATG TTT TTG GAA CGC CAG AG	0.5 µM		

**Table 4:** Species identification of isolates obtained from each farm.

Origin	Number of Isolates (Percentages)		
	*E. faecalis*	*E. faecium*	Total
Farm A (one-day-old chicks)	30 (100%)	0	30
Farm A (pullets)	12 (20%)	48 (80%)	60
Farm A (adult hens)	5 (8.3%)	55 (91.7%)	60
Farm B (adult hens)	20 (11.1%)	160 (88.9%)	180
Total (%)	67 (20.3)	263 (68.7%)	330

**Table 5:** Percentage of resistant isolates for each antibiotic tested.

Farm	Species	Antibiotic								
		E	AMP	CIP	VA	C	CN	S	TE	QD
A	*E. faecalis* (47)	59.6%	0%	59.6%	0%	59.6%	0%	0%	78.7%	ND
		(28)	(0)	(28)	(0)	(28)	(0)	(0)	(37)	ND
	*E. faecium* (103)	58.3%	1.0%	5.8%	0%	0%	0%	0%	95.2%	6.8%
		(60)	(1)	(6)	(0)	(0)	(0)	(0)	(98)	(7)
B	*E. faecalis* (20)	10%	0%	0%	0%	0%	0%	0%	65%	ND
		(2)	(0)	(0)	(0)	(0)	(0)	(0)	13	ND
	*E. faecium* (160)	16.9%	0%	0.6%	0%	0%	0%	0%	86.9%	0.6%
		(27)	(0)	(1)	(0)	(0)	(0)	(0)	(139)	(1)

QD only tested for *E. faecium*. E = erythromycin; AMP = ampicillin; CIP = ciprofloxacin; VA = Vancomycin; C = chloramphenicol; CN = gentamicin; S = streptomycin; TE = tetracycline; QD = quinopristin/dalfopristin. Numbers in parentheses represent the number of isolates. ND: Not determined.

**Table 6:** Antibiotic resistance profiles observed in *E. faecalis* and *E. faecium* with one or more antibiotic resistances.

Profile	Farm A		Farm B	
	*E. faecalis* (47)	*E. faecium* (103)	*E. faecalis* (20)	*E. faecium* (160)
E	0	1% (1)	0	0.7% (1)
TE	24.3% (9)	40% (40)	84.6% (11)	80.7% (113)
E-TE	0	45% (45)	15.5% (2)	17.1% (24)
E-AMP	0	1% (1)	0	-
E-QD-TE *	-	7% (7)	-	0.7% (1)
E-TE-CIP	0	6% (6)	0	0.7% (1)
E-TE-C-CIP	75.7% (28)	-	0	-

E = erythromycin; TE = tetracycline; AMP = ampicillin; QD = quinopristin/dalfopristin; CIP = ciprofloxacin. Numbers in parentheses represent the number of isolates. * QD susceptibility was not tested for *E. faecalis* isolates.

**Table 7:** Prevalence of the antibiotic resistance genes detected in isolates resistant to erythromycin and tetracycline.

Farm	Species (Nº of Isolates)	*erm*A	*erm*B	*tet*K	*tet*L	*tet*M	*tet*O	*tet*S
Farm A	*E. faecalis* (28)	28 (100%)	28 (100%)	2	28 (100%)	28 (100%)	0	0
	*E. faecium* (58)	3	55 (94.8%)	50 (86.2%)	53 (91.4%)	55 (94.8%)	0	1
Farm B	*E. faecalis* (2)	0	2	1	1	1	1	1
	*E. faecium* (26)	0	9	22 (84.6%)	23 (88.5%)	18 (69.2%)	0	0

## Data Availability

The original contributions presented in this study are included in the article. Further inquiries can be directed to the corresponding author.

## Supplementary Materials

The following supporting information can be downloaded at: https://www.mdpi.com/article/10.3390/vetsci13080795/s1. Table S1: Origin, species identification, antimicrobial resistance phenotypes, and resistance gene profiles of *Enterococcus faecalis* and *Enterococcus faecium* isolates recovered from two laying hen farms. Table S2: Cultural characteristics and PCR identification results of presumptive *Enterococcus* isolates recovered from laying hen farms. Table S3: CLSI M100-S25 interpretive criteria used for antimicrobial susceptibility testing of *Enterococcus* spp. Tables S4: Statistical analysis of results. Figure S1: *erm*B gene detection by PCR in different *Enterococcus* spp. isolates. Figure S2: *tet*M gene detection by PCR in different *Enterococcus* spp. isolates.
