# Supplementary material for: Antimicrobial Resistance and Species Dynamics of Enterococcus faecalis and Enterococcus faecium in Breeding Hens: Phenotypic and Genotypic Characterization
Source: Vet Sci. 2026 Aug 9;13(8):795. doi: 10.3390/vetsci13080795 (PMC13517455; doi:10.3390/vetsci13080795)
Supplement: Supplementary file 1 [file vetsci-13-00795-s001.zip › Supplementary Material Table S4.pdf]

Tables S4: Statistical analysis of results

Table S4a. Comparison of antibiotic resistance (AMR) rates between farms for *E. faecalis* and *E. faecium*

Species	AMR	Farm A n/N[1]	Farm B n/N[1]	Statistical significance (p-value)	OR (95% CI)
E. faecalis	E	28/47	2/20	Yes (0.0002)	13.26 (2.75-63.93)
E. faecalis	TE	37/47	13/20	No (0.357)	1.99 (0.63-6.32)
E. faecalis	CIP	28/47	0/20	Yes (<0.0001)	59.92 (3.42-1051)
E. faecium	E	60/103	27/160	Yes (<0.0001)	6.87 (3.89-12.15)
E. faecium	TE	98/103	139/160	Yes (0.034)	2.96 (1.08-8.12)
E. faecium	CIP	6/103	1/160	Yes (0.016)	9.84 (1.17-82.93)
E. faecium	QD	7/60	1/160	Yes (0.0005)	21.00 (2.53-174.64)

AMR: Antibiotic resistance

[1] number of isolates presenting resistance /number of total isolates

Table S4b. Comparison of antibiotic resistance (AMR) between *E. faecalis* and *E. faecium* (farms pooled)

AMR	*E. faecalis* n/N[1]	*E. faecium* n/N[1]	Statistical significance (p-value)	OR (95% CI)
E	30/67	87/263	No (0.086)	1.64 (0.95-2.83)
TE	50/67	237/263	Yes (0.0018)	0.32 (0.16-0.64)
CIP	28/67	7/263	Yes (<0.0001)	26.26 (10.74-64.20)

AMR: Antibiotic resistance

[1] number of isolates presenting resistance /number of total isolates from both farms

Table S4c. Comparison of antibiotic resistance (AMR) between Farm A and Farm B (species pooled)

AMR	Farm A n/N[3]	Farm B n/N[3]	Statistical significance (p-value)	OR (95% CI)
E	88/150	29/180	Yes (<0.0001)	7.39 (4.42-12.35)
TE	135/150	152/180	0.143	1.66 (0.85-3.24)
CIP	34/150	1/180	Yes (<0.0001)	52.47 (7.08-388.55)

[3] Number of resistant isolates/total number of isolates from both species.

Table S4d. Comparison of antibiotic resistance gene (ARG) prevalence in *E. faecium* isolates between farms

**Species**	**ARG**	**Farm A n/N**[4]	**Farm B n/N**[4]	**Statistical significance (p-value)**	**OR (95% CI)**
E. faecium	*erm*B	55/58	9/26	Yes (<0.0001)	34.63 (8.41-142.60)
E. faecium	*tet*K	50/58	22/26	No (1.000)	1.14 (0.31-4.17)
E. faecium	*tet*L	53/58	23/26	No (0.698)	1.38 (0.30-6.28)
E. faecium	*tet*M	55/58	18/26	Yes (0.003)	8.15 (1.95-34.04)

ARG: Antibiotic resistance gene
[2]nº of isolates presenting the resistance gene/number of total isolates resistant to E and TE

Table S4e. Comparison of antibiotic resistance profiles between farms for *E. faecalis* and *E. faecium*

**Species**	**Profile**	**Farm A n/N**[5]	**Farm B n/N**[5]	**Statistical significance (p-value)**	**OR (95% CI)**
E. faecalis	TE	9/47	11/20	Yes (0.0073)	0.19 (0.06-0.61)
E. faecium	TE	40/103	113/160	Yes (<0.0001)	0.26 (0.16-0.45)
E. faecium	E-TE	45/103	24/160	Yes (<0.0001)	4.40 (2.45-7.88)

[5] number of isolates presenting each pattern/number of total isolates

Table S4f. Comparison of antibiotic resistance profiles between *E. faecalis* and *E. faecium* (farms pooled)

**Profile**	***E. faecalis*** **n/N**[6]	***E. faecium*** **n/N**[6]	**Statistical significance (p-value)**	**OR (95% CI)**
TE	20/67	153/263	Yes (0.00012)	0.32 (0.18-0.58)
E-TE	2/67	69/263	Yes (<0.0001)	0.085 (0.02-0.36)

[6] number of isolates presenting each pattern/number of total isolates from both farms

<u>Methodology</u>:

Statistical comparisons were performed using Fisher's exact test. Odds ratios (OR) and 95% confidence intervals (CI) were calculated from 2 × 2 contingency tables. Statistical significance was not estimable when all isolates in both groups were positive for the analysed gene.

The statistical results in gene prevalence among *E. faecalis* isolates was not determined, as the number of resistant isolates available for molecular characterization from Farm B was very small (n = 2). This limitation may have reduced the ability to detect true between-farm differences.

Statistical analyses were not performed when one comparison group contained no positive isolates, as this resulted in unstable effect estimates and biologically uninformative confidence intervals.
