# Supplementary material for: Antimicrobial Resistance and Species Dynamics of Enterococcus faecalis and Enterococcus faecium in Breeding Hens: Phenotypic and Genotypic Characterization
Source: Vet Sci. 2026 Aug 9;13(8):795. doi: 10.3390/vetsci13080795 (PMC13517455; doi:10.3390/vetsci13080795)
Supplement: Supplementary file 1 [file vetsci-13-00795-s001.zip › Supplementary Material Tables S3.pdf]

Table S3a. CLSI M100-S25 interpretive criteria used for antimicrobial susceptibility testing of *Enterococcus* spp.

**Antimicrobial agent**	**Disk content**	**Susceptible (mm)**	**Intermediate (mm)**	**Resistant (mm)**
Ampicillin	10 µg	≥17	NA	≤16
Vancomycin	30 µg	≥17	15-16	≤14
Erythromycin	15 µg	≥23	14-22	≤13
Tetracycline	30 µg	≥19	15-18	≤14
Ciprofloxacin	5 µg	≥21	16-20	≤15
Chloramphenicol	30 µg	≥18	13-17	≤12
Quinupristin/dalfopristin	15 µg	≥19	16-18	≤15

Table S3b. CLSI M100-S25 interpretive criteria used for antimicrobial susceptibility testing of *Enterococcus* spp. for aminoglycosides (High-level resistance)

**Antimicrobial agent**	**Disk content**	**Susceptible**	**Non conclusive**	**Resistant**
Gentamicin (high level)	120 µg	≥ 10 mm	7-9 mm	≤ 6 mm
Streptomycin (high level)	300 µg	≥ 10 mm	7-9 mm	≤ 6 mm
